# Cystoid macular edema associated with omidenepag isopropyl in a phakic eye with an implantable collamer lens: a case report

**DOI:** 10.1186/s12886-023-03091-0

**Published:** 2023-07-26

**Authors:** Byung-Jin Bae, Eun Min Kang, Sang Yeop Lee

**Affiliations:** 1 Department of Ophthalmology, Yongin Severance Hospital, Yonsei University College of Medicine, 363 Dongbaekjukjeon-daero, Giheung-gu, Yongin-si, Gyeonggi-do 16995 Korea; 2 B&VIIT Eye Center, Seoul, South Korea

**Keywords:** Implantable collamer lens, Omidenepag isopropyl, Cystoid macular edema, Phakic eye, Case report

## Abstract

**Background:**

Cystoid macular edema is a known complication of omidenepag isopropyl usage. Omidenepag isopropyl is a selective prostanoid EP2 receptor agonist, and its association with macular edema has mainly been identified in pseudophakic eyes. Herein, we report a case of cystoid macular edema caused by omidenepag isopropyl use in a phakic eye with an implantable collamer lens.

**Case presentation:**

A 38-year-old woman was diagnosed with left eye glaucoma and prescribed omidenepag isopropyl. She had undergone bilateral implantation of implantable collamer lenses approximately 12 years prior to the glaucoma diagnosis. After 9 months of using omidenepag isopropyl, she presented with blurred vision in the left eye; swept source optical coherence tomography revealed cystoid macular edema in this eye. Omidenepag isopropyl usage was discontinued, and bromfenac sodium hydrate was administered twice daily instead. After 2 months, the patient’s visual discomfort was completely ameliorated. Additionally, an optical coherence tomography examination confirmed that the macula had normalized.

**Conclusions:**

We report a case of cystoid macular edema development after omidenepag isopropyl use in a patient with glaucoma who had undergone bilateral implantable collamer lens implantation. This case shows that the possibility of cystoid macular edema occurrence should be considered when omidenepag isopropyl is used, even in phakic eyes, after the insertion of implantable collamer lenses.

## Background

Omidenepag isopropyl (OMDI), a selective non-prostaglandin prostanoid EP2 receptor agonist, has been recently used for the treatment of glaucoma. OMDI induces a reduction in the intraocular pressure by acting on both the conventional and unconventional aqueous humor outflow pathways [1]. The advantage of using OMDI is the absence of the prostaglandin-associated syndrome even when administered daily [2–4]. This has led to its widespread use in the treatment of glaucoma in patients with cosmetic sensitivities or those who require anti-glaucoma medication for only one eye. However, OMDI should be used with caution as it can lead to macular edema. Most cases of macular edema so far have been reported in pseudophakic eyes. However, additional reports have emerged of macular edema in phakic eyes [5, 6]. In this report, we describe the case of a patient with open-angle glaucoma with implantable collamer lenses (ICLs) who developed cystoid macular edema (CME) after using an OMDI ophthalmic solution.

## Case presentation

**Fig. 1:**
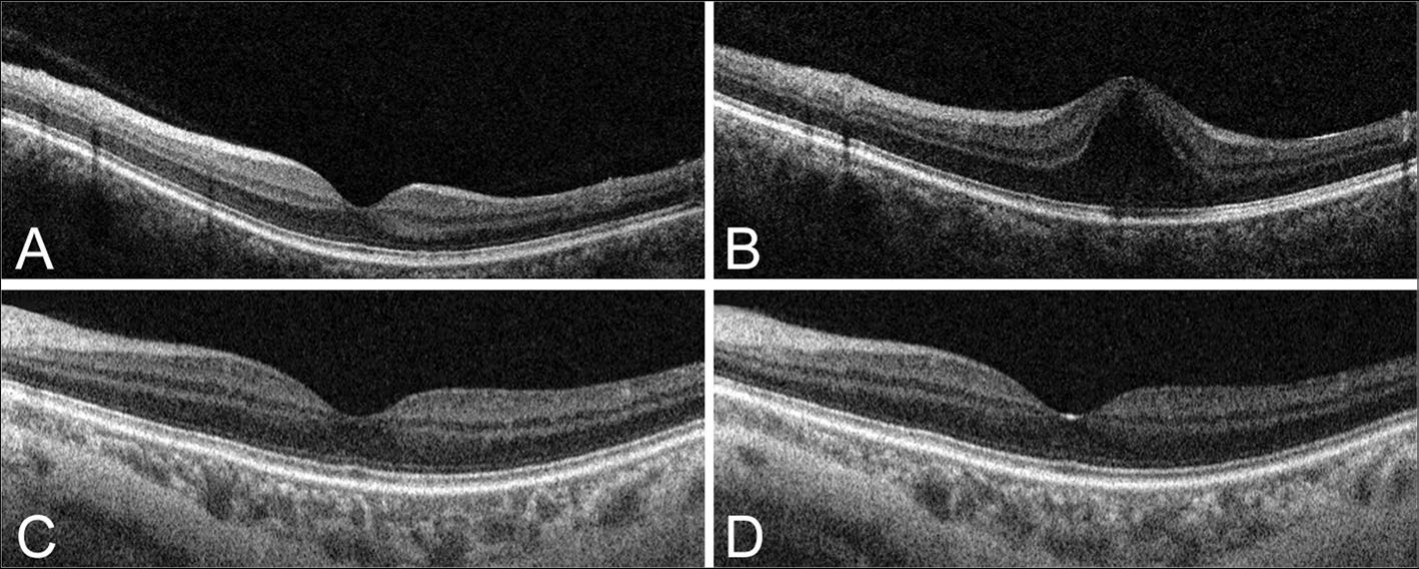
Swept source optical coherence tomography images of the left eye. **A** Normal macular findings before omidenepag isopropyl use. **B** Cystoid macular edema after 9 months of omidenepag isopropyl use. **C** One month after omidenepag isopropyl discontinuation. **D** Two months after omidenepag isopropyl discontinuation

On April 30, 2009, a 38-year-old woman underwent ICL implantation in both eyes for correction of a refractive error. Twelve years later, slit lamp examination and gonioscopy confirmed the presence of an open-angle structure in the left eye, with no other abnormalities in the anterior eye segment (including the cornea, trabecular meshwork, and iris). Furthermore, glaucomatous changes in the optic disc and retinal nerve fiber layer as well as visual field defects were observed. Thus, she was diagnosed with open-angle glaucoma of the left eye. The patient had no history of any other ophthalmic diseases, systemic diseases (such as systemic hypertension or diabetes mellitus), and treatments (except for a bilateral laser iridotomy for ICL implantation). At the time of glaucoma diagnosis, the best corrected visual acuity (BCVA) was 20/20, and the intraocular pressure in both eyes (determined using a Goldmann applanation tonometer) was 15 mmHg. Furthermore, swept source optical coherence tomography (SS-OCT) revealed a normal macula in the left eye (Fig. 1A). Considering that topical anti-glaucoma medications should only be administered to one eye in young patients, an OMDI ophthalmic solution was prescribed. However, the patient complained of visual discomfort in the left eye following approximately 9 months of solution usage, although the BCVA did not deteriorate. The presence of CME was confirmed using SS-OCT (Fig. 1B). The OMDI solution was discontinued immediately, and topical bromfenac sodium hydrate was administered to the left eye (twice daily) to improve the CME instead. One month after OMDI discontinuation, SS-OCT revealed improvement in the CME (Fig. 1C). The patient’s visual discomfort disappeared 2 months later with normalization of the macula (Fig. 1D). After confirming the absence of CME recurrence through an additional 1-month follow-up, the patient started using a topical beta blocker for the treatment of glaucoma in the left eye.

## Discussion and conclusions

CME is characterized by increased retinal thickness and cystic changes in the outer plexiform and inner nuclear layers. It is accompanied by a loss of the superficial and deep capillary plexus [7]. An important factor in the pathogenesis of CME is damage to the blood–retinal and blood–aqueous barriers [8, 9]; inflammation is an important factor involved in the disruption of these barriers. Therefore, conditions that lead to inflammation, such as intraocular surgery or use of proinflammatory medications, can increase the likelihood of CME development [7]. The patient in the present case had ICLs implanted in both eyes. Inflammation caused by friction between the ICL and the posterior iris surface or lens as well as vitreous changes induced by ICL implantation have been implicated in the post-implantation occurrence of CME [10]. Although a case of CME that occurred after ICL implantation has been reported previously [11], it differs from the one presented in this report in that the previous case occurred 2 months after ICL implantation and the patient therein underwent ICL repositioning after implantation.

Considering that the condition of the patient in this case remained stable after ICL implantation, we believe that their OMDI use was most likely associated with CME occurrence. As with conventional topical prostaglandin analogs [3, 7, 12], the possibility of CME occurrence arises from OMDI being an EP2 receptor agonist related to prostaglandin E2 [7]. In Japan, phase II and III clinical studies found that macular edema occurred in 5.2% of the study population after OMDI ophthalmic solution usage; all affected cases involved pseudophakic eyes [2]. Thus, in clinical practice, OMDI is not typically used for pseudophakic and aphakic eyes. However, macular edema has been reported with OMDI use even in phakic eyes. In a post-marketing observational interim analysis that included only phakic eyes, four cases of macular edema were detected among 1862 patients [6]. Among these four patients (all aged approximately 60 years), only one was treated with OMDI alone; furthermore, they presented with a history of epiretinal membrane (*n* = 1), lattice degeneration with a history of retinal photocoagulation (*n* = 1), and a history of trabeculectomy (*n* = 1). Similarly, a case of CME development after OMDI use has been reported in a 59-year-old patient with primary angle closure glaucoma and a history of laser iridotomy [5]; the patient had a phakic eye and took latanoprost before the iridotomy.

Therefore, although rare, CME can occur in phakic eyes following OMDI use. The case reported herein is significant because unlike previously reported cases, it involved CME that developed after topical administration of OMDI to the phakic eye of a relatively young patient who had undergone ICL implantation. In addition, the patient had no history of using conventional prostaglandin analogs prior to OMDI use; she further had no history of other ophthalmic diseases as well. The laser iridotomy required for ICL implantation [13] or the insertion of the ICL itself may have created an inflammation-prone environment that subsequently led to the breakdown of the blood–aqueous or blood–retinal barrier. Although additional research is needed to verify the cause of CME in the present case, clinicians must be aware of the fact that CME can occur as a result of OMDI use after ICL implantation even in phakic eyes.

## Data Availability

The datasets used and/or analyzed during the current study are available from the corresponding author on reasonable request.

## Abbreviations

OMDI: Omidenepag Isopropyl
ICL: Implantable Collamer Lens
CME: Cystoid Macular Edema
BCVA: Best Corrected Visual Acuity
SS-OCT: Swept Source Optical Coherence Tomography
